# Bibliometric analysis and visualization of literature on assisted reproduction technology

**DOI:** 10.3389/fmed.2022.1063040

**Published:** 2022-12-01

**Authors:** Fanchao Meng, Sheng Deng, Lu Wang, Yumei Zhou, Mengjie Zhao, Haibin Li, Dong Liu, Guojing Gao, Xiaoxing Liao, Jisheng Wang

**Affiliations:** ^1^Urology Surgery, The Third Affiliated Hospital of Beijing University of Chinese Medicine, Beijing, China; ^2^Department of Andrology, Shunyi Hospital, Beijing Hospital of Traditional Chinese Medicine, Beijing, China; ^3^Department of Surgery, Beijing Xuanwu Traditional Chinese Medicine Hospital, Beijing, China; ^4^Department of Gynaecology, The Third Affiliated Hospital of Beijing University of Chinese Medicine, Beijing, China; ^5^Department of Andrology, Dongzhimen Hospital, Beijing University of Chinese Medicine, Beijing, China

**Keywords:** assisted reproduction technology, bibliometric analysis, CiteSpace, VOSviewer, visualization

## Abstract

**Introduction:**

Assisted reproductive technology (ART) is a method that uses various techniques to process sperm or ova. Assisted reproductive technology involves removing ova from a woman's ovaries, combining them with sperm in the laboratory, and returning them to the woman's body or donating them to another woman.

**Methods:**

Based on the web of science core collection database, we firstly analyzed the quantity and quality of publications in the field of ART, secondly profiled the publishing groups in terms of country, institution, author's publication and cooperation network, and finally sorted out and summarized the hot topics of research.

**Results:**

In total, 6,288 articles on ART were published between 2001 and 2022 in 1,013 journals. Most of these published articles represent the global research status, potential hotspots and future research directions. Publications and citations of research on assisted reproductive technology have steadily increased over the past few decades. Academic institutions in Europe and the United States have been leading in assisted reproductive technology research. The countries, institutions, journals, and authors with the most published articles were the United States (1864), Harvard Univ (108), Fertility and Sterility (819), and Stern, Judy E. (64). The most commonly used keywords are Assisted reproductive technology (3303) and *in-vitro* Fertilization (2139), Ivf (1140), Pregnancy (1140), Women (769), Intracytoplasmic Sperm injection (644), In Fertilization (632), Risk (545), and Outcome (423).

**Conclusion:**

Frozen embryo transfer, intracytoplasmic sperm injection, and *in vitro* fertilization are the main research topics and hotspots in the field of assisted reproductive technology.

## Introduction

The World Health Organization (WHO) states that infertility is the failure to conceive after 12 months of having unprotected sexual intercourse. According to WHO, infertility affects about 15 percent of couples worldwide, where the influence of male factors can be found in 30–50% of cases (1). Assisted reproductive technology (ART) involves removing eggs from a woman's ovaries, combining them with sperm in the laboratory, and returning them to the woman's body or donating them to another woman. The techniques used in ART include artificial insemination (AI), *in vitro* fertilization-embryo transfer (IVF-ET) and related technologies, such as intracytoplasmic sperm injection (ICSI), preimplantation genetic screening (PGS), *in vitro* oocyte maturation (IVM), assisted hatching (AH) and oocyte vitrification and freezing technology. Recent data from the European Society for Human Reproduction and Embryology (ESHRE) show that ART (including all treatment modalities) pregnancy rates in 39 countries range from 17.1 to 53.1%, and live birth rates range from 7.9 to 37.8% (2), although the increase has been modest.

The term bibliometric was coined by Alan Pritchard in 1969 (3). Bibliometric analysis is a powerful tool for exploring and analyzing large volumes of scientific data (4, 5). CiteSpace and VOSviewer are the most commonly used visual processing tools for bibliometric analysis of co-word, co-citation and literature coupling (6).

Based on the advantages of clustering technology and map presentation, the research trend of a specific field is analyzed and displayed in the form of a multivariate comprehensive visual knowledge map (7, 8). Bibliometric software helps visually present and analyze the literature related to assisted reproduction. This study aims to systematically analyze and visualize ART-related publications through bibliometrics, and to reveal identified topics, hotspots, and knowledge gaps in related fields.

## Materials and methods

### Ethics statement

The study needed no approval from the institutional review board because it involved the analysis of retrieved scientific measurement data from the Web of Science database (WOS), and no human subjects were involved.

### Sources and collection

Web of Science (WOS) database is the most commonly used and acceptable database in scientific or bibliometric research because it contains nearly 9,000 of the world's most prestigious high-impact journals and more than 12,000 academic conferences. The published articles in WOS provide a comprehensive overview of the world's research results in science, technology, medicine and other fields (9, 10).

This study searched WOS for information on assisted reproductive technology within 1 day to ensure no data were updated. The search timeframe was set between 2001.01.01 and 2022.08.25, and the retrieval date is 2022.08.26. The search was conducted by selecting “WOS Core Collection” with the topic word “Assisted Reproductive Technology” and the article type “Article” and “Review.” Then the retrieved files were exported in the “Plain Text File” format, and “Full Record and Cited References” was selected for “Record Content.”

The search query string was described as follows: Results for “assisted reproductive technology” (Topic) and Article or Review Article (Document Types) and Book Chapters (Exclude–Document Types).

### Bibliometric analysis and software

CiteSpace software (Drexel University, Philadelphia, Pennsylvania, USA) is a freely available Java application widely used to visualize and analyze trends and patterns in the scientific literature (11). CiteSpace software was designed by Dr. Chen Chaomei in 2004 (7). CiteSpace to scientometrics, data and information visualization technology as the foundation, through the analysis of the potential knowledge of literature, regularity and distribution, and present knowledge structure. This study used CiteSpace software for keyword clustering and salient word analysis.

On the other hand, the VOSviewer is a literature measurement analysis software for drawing knowledge. Common words can be used in the literature analysis, total cited and coupling analysis, and visualization display (12). This study used the VOSviewer to visualize countries/regions, authors, institutional collaborations, cited journals, keyword co-occurrence and construct density maps.

This study aims to describe all literature characteristics, including country/institution, journal, highly cited articles, cluster network of co-cited references, and most frequently cited keywords. In addition to noun phrases extracted from article titles and abstracts, burst detection was applied to the keywords of publications in the article collection assigned to the citation extension.

## Results

### Time trends in publications and citations

**Figure 1:**
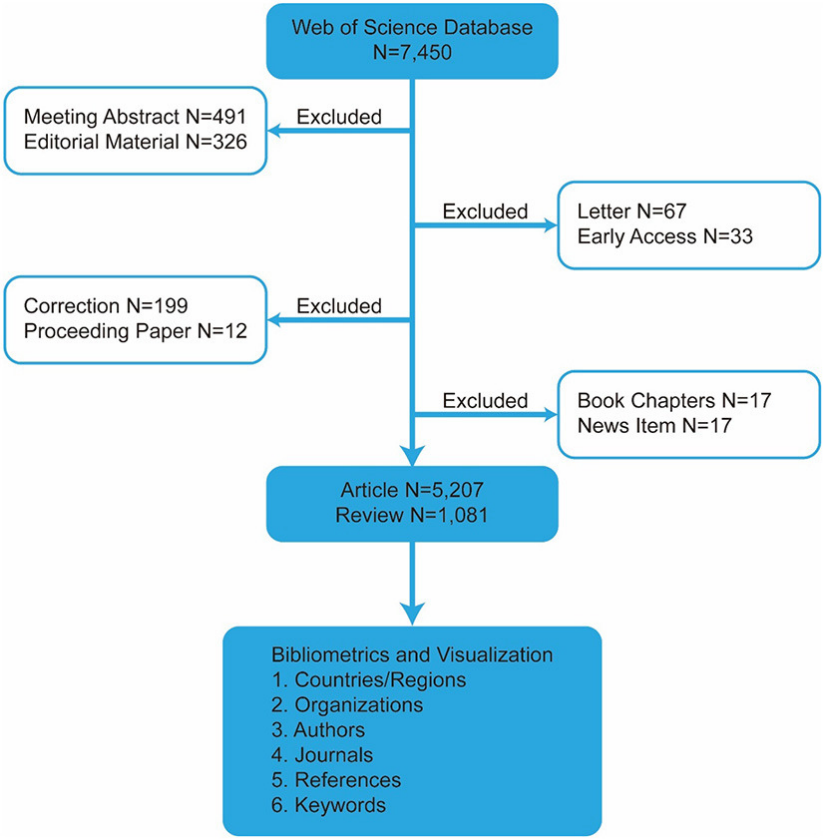
Flowchart of the searching stagey in the study.

**Figure 2:**
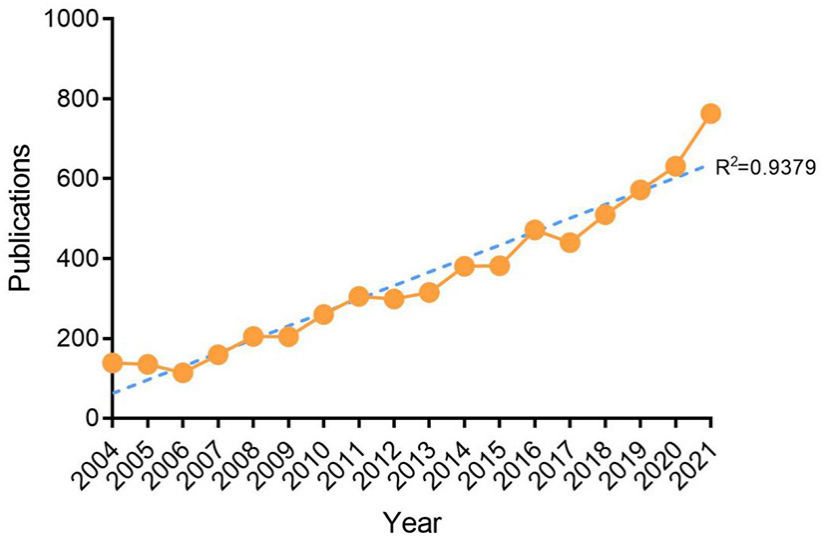
Annual trends of global publications.

The number of annual publications is important in the development of scientific research since it reflects the growth of knowledge in this field. As of August 25, 2022, 6,288 articles on ART had been published, as shown in Figure 1, and the number of papers published per year is shown in Figure 2. Although the overall trend of published articles is increasing, the results found that the trend fluctuated in some years. Nonetheless, the study found that the number of publications per year can be divided into three phases: phase one, from 2004 to 2008, when the average annual number of publications was between 139 and 205. Phase two was from 2009 to 2016, with an average number of publications between 204 and 472 per year, and phase three was from 2017 to 2021, with an average annual number of publications between 440 and 763. In addition, the study found that the knowledge of ART showed a linear growth trend (R^2^ = 0.9379), reflecting the increasing research interest in this field.

### Analysis of top productive countries/regions

**Figure 3:**
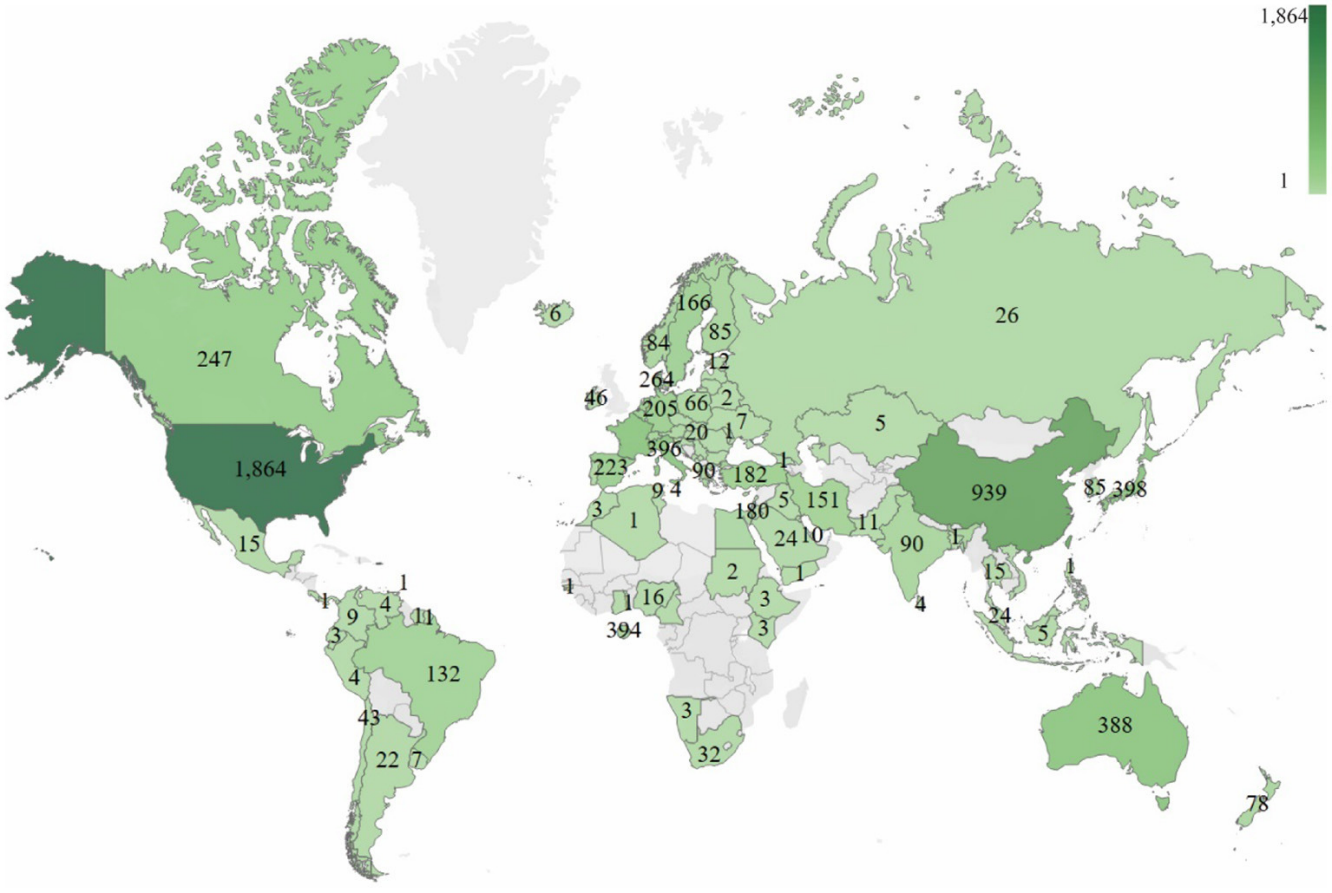
Analysis of the research trends based on the origin of publications globally.

In total, 69 countries/regions have published papers on ART. The top 10 countries with outstanding contributions to publications on ART are the USA (1864), China (862), Japan (398), Italy (395), France (394), England (393), Australia (388), Denmark (263), Canada (247) and the Netherlands (223), as shown in Figure 3, Table 1.This study determined that the size of the node is determined by the number of publications (the larger the number, the larger the node) and that the same colors represent the same clusters. On the other hand, the lines between nodes represent the alignment between countries/regions (the stronger the partnership, the wider the boundaries), and the number of total link strengths reflects the combined strength between countries/regions. These results show that the USA has the largest number of publications (1864, 29.61%), the highest number of citations (61,510), and the link strength (961). The results in Table 2 show that the USA has the highest number of citations (61510), followed by Australia (16484), England (15591), the Netherlands (12544) and France (12541). These results indicate that these countries have a great influence on ART research.

**Table 1:** The top 10 countries contributing to ART research.

**Rank**	**Country**	**Documents**	**Citations**	**Total link strength**
1	USA	1864	61510	961
2	China	862	11144	235
3	Japan	398	9525	168
4	Italy	395	10903	567
5	France	394	12541	498
6	England	393	15591	648
7	Australia	388	16484	441
8	Denmark	263	9554	464
9	Canada	247	9257	222
10	Netherlands	223	12544	418

**Table 2:** Number of citations of publications on ART for the top 10 countries.

**Rank**	**Country**	**Documents**	**Citations**	**Total link strength**
1	USA	1864	61510	961
2	Australia	388	16484	441
3	England	393	15591	648
4	Netherlands	223	12544	418
5	France	394	12541	498
6	China	862	11144	235
7	Italy	395	10903	567
8	Belgium	196	10090	483
9	Denmark	263	9554	464
10	Japan	398	9525	168

**Figure 4:**
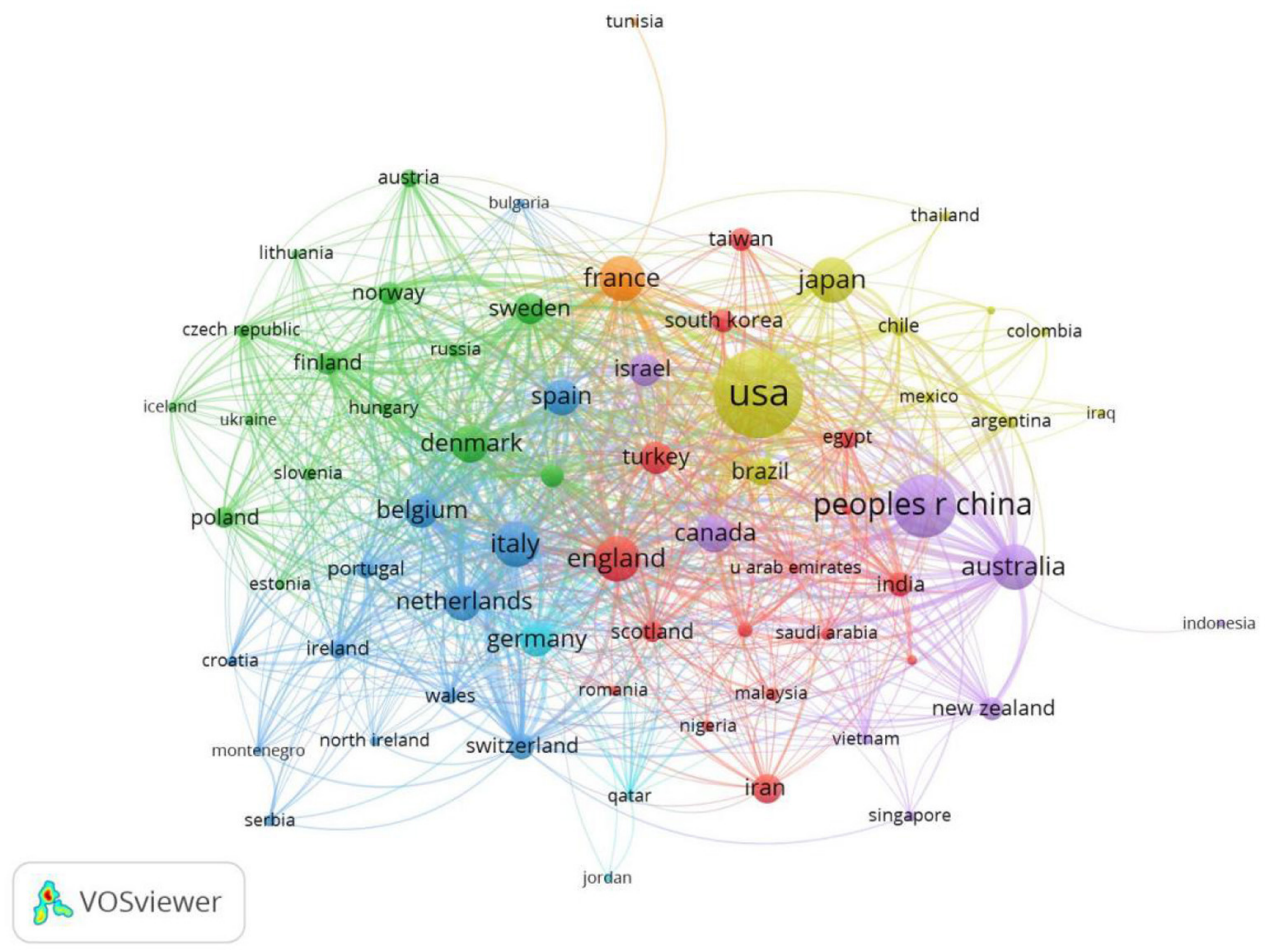
Co-occurrence map of Countries/Regions. The size of the nodes represents the number of articles, the thickness of the curve represents the strength of collaboration, and the colors represent different collaboration groups.

VOSviewer was used to analyze cooperation across countries, with lines between nodes indicating co-authorship between countries and thicker lines indicating stronger cooperation. The results in Figure 4 show that the USA, China, Australia and England had stronger cooperation and other countries had a weaker cooperation.

### Contributions of top organizations

**Figure 5:**
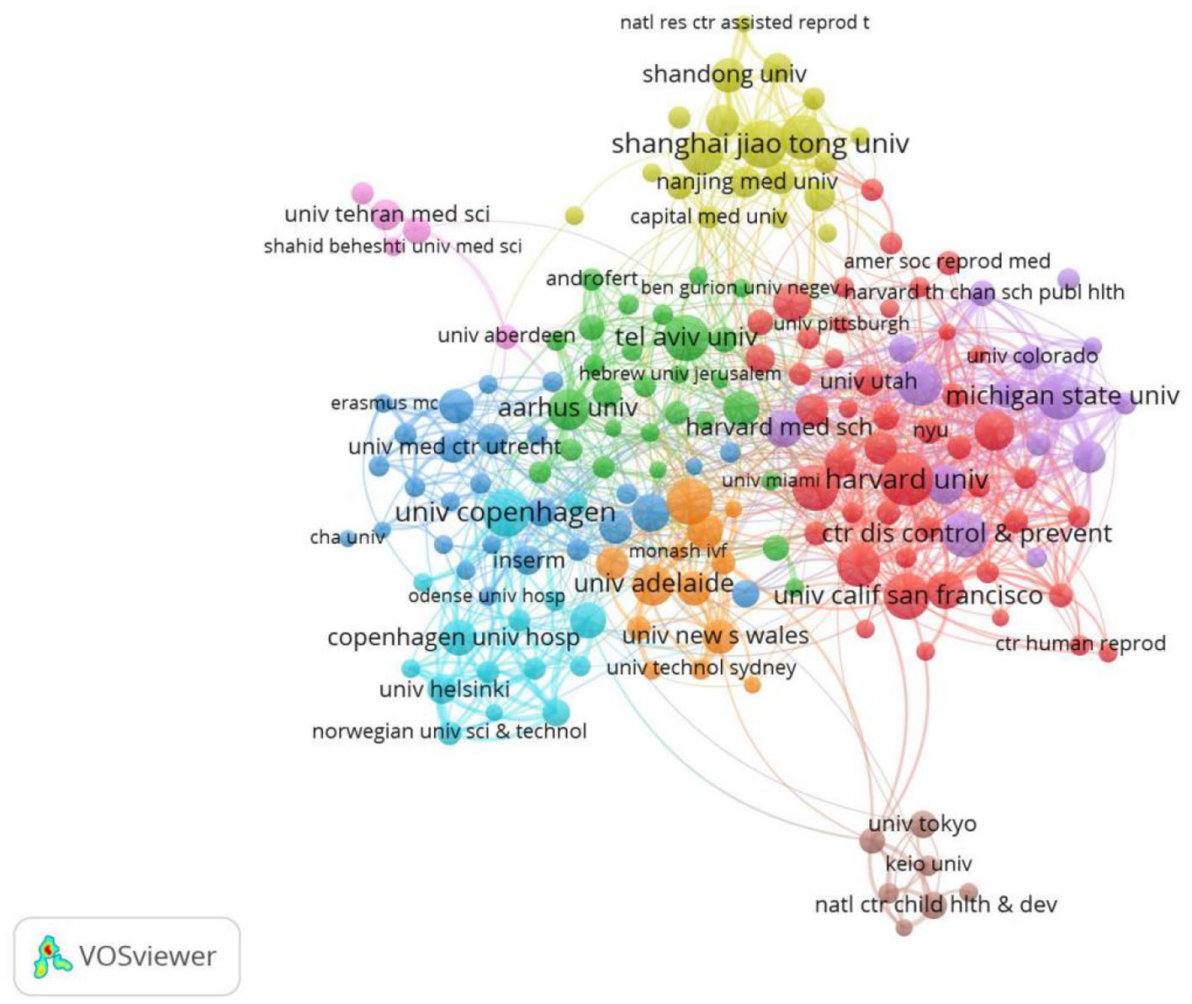
Co-occurrence map of Institutions. The size of the nodes represents the number of articles, the thickness of the curve represents the strength of collaboration, and the colors represent different collaboration groups.

In total, 5,754 institutions published papers on ART, and the top five institutions with outstanding contributions to ART research were Harvard Univ (108), Univ Copenhagen (90), Shanghai Jiao Tong Univ (90), Tel Aviv Univ (88) and Ctr DisControl & Prevent (88), as shown in Table 3. These results indicate that Harvard Univ has the largest number of publications (108, 1.72%), the highest citation (4468), and the link strength of 248. The map has 176 terms, 9 clusters, and 1,627 links for a total link strength of 4,241. Each node represents a different institution. The size of the node is determined by the number of publications (the larger the number, the larger the node), and the same colors represent the same clusters. Boundaries between nodes represent a collaboration between organizations (the stronger the partnership, the wider the boundaries), and the number of link strengths reflect the aggregate strength between institutions. The visual map shows that 176 institutions cooperate both within and between clusters, and the top three institutions with the highest total link strength are Michigan State Univ (*n* = 375), Harvard Univ (*n* = 248), Brigham & Women's Hos (*n* = 232). The results in Table 4 show that the top five institutions with the highest number of citations include Harvard UNIV (4468), Ctr DIS Control & Prevnt (4020), UNIV Adelaide (3843), UNIV New S Wales (3756) and UNIV, Calif SAN Francisco (3674). Harvard UNIV, UNIV Copenhagen and Shanghai Jiao Tong Univ are at the center of the partnership. On the other hand, the results show that most institutions are fragmented and lack cooperation. The overall density of the network is low (density = 0.0139), mainly conducted in institutions in Europe and the United States, as shown in Figure 5.

**Table 3:** The top 10 institutions contributing to publications on ART.

**Rank**	**Organization**	**Documents**	**Citations**	**Total link strength**
1	Harvard Univ	108	4468	248
2	Univ Copenhagen	90	2839	191
3	Shanghai Jiao Tong Univ	90	1738	166
4	Tel Aviv Univ	88	1192	148
5	Ctr Dis Control & Prevent	88	4020	136
6	Michigan State Univ	86	2715	375
7	Monash Univ	85	2446	227
8	Univ Calif San Francisco	84	3674	198
9	Yale Univ	83	2880	165
10	Brigham & Women's Hos	79	2088	232

**Table 4:** Number of citations by the top 10 institutions contributing to ART research.

**Rank**	**Organization**	**Documents**	**Citations**	**Total link strength**
1	Harvard Univ	108	4468	248
2	Ctr Dis Control & Prevnt	88	4020	136
3	Univ Adelaide	70	3843	118
4	Univ News Wales	52	3756	134
5	Univ Calif San Francisco	85	3674	198
6	Univ Auckland	59	3380	109
7	Univ Amsterdam	52	3372	108
8	Yale Univ	84	2880	165
9	Univ Med Ctr Utrecht	43	2851	118
10	Univ Copenhagen	90	2839	191

### Analysis of authors and co-cited authors

**Figure 6:**
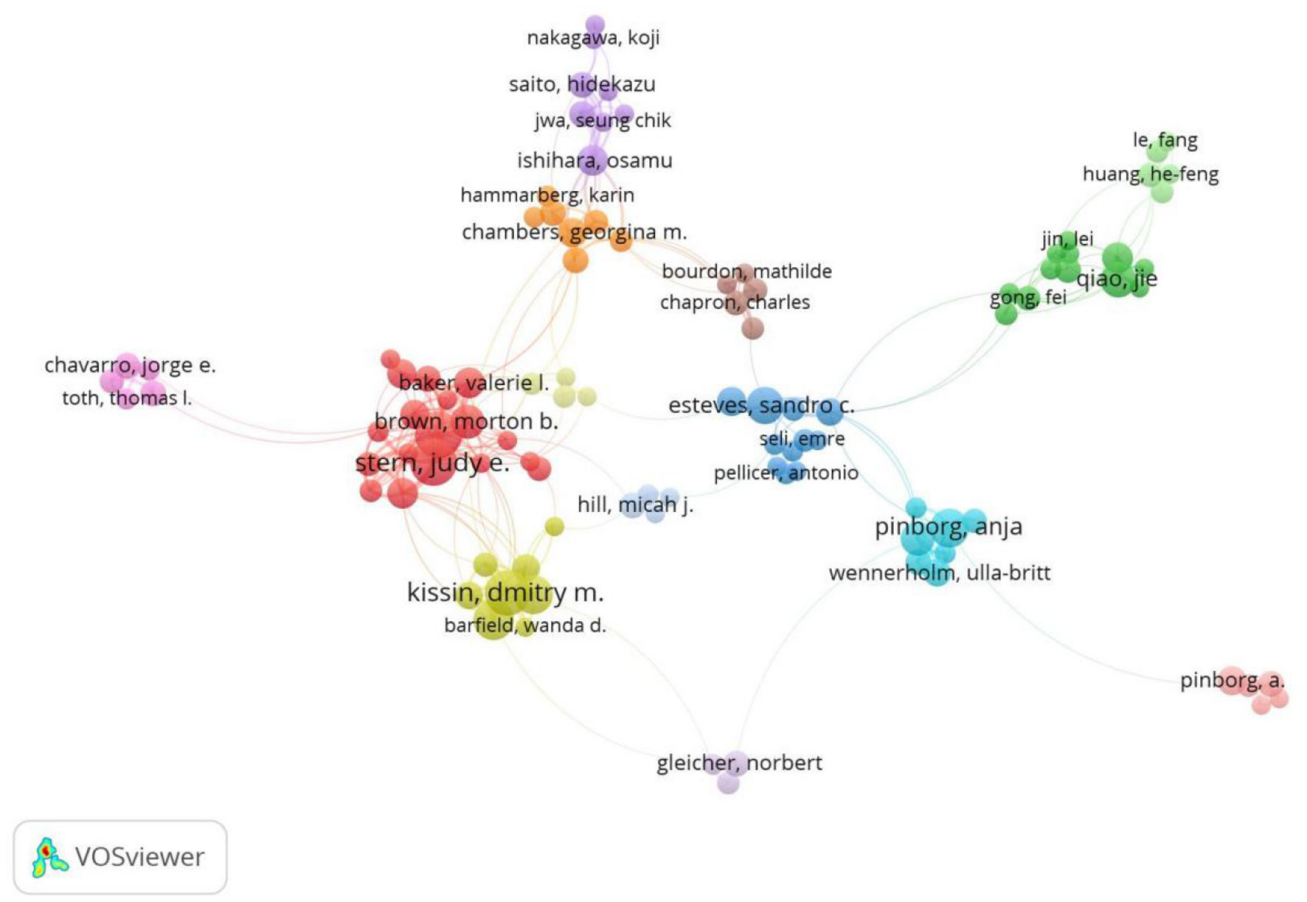
CiteSpace visualization map of authors involved in assisted reproduction. Nodes represent authors (the larger the circle, the more publications), the lines between nodes represent the cooperation between two authors of the same article (the wider the line, the more frequent the cooperation), and the color in the node represents the year.

Author co-occurrence analysis identifies the core authors and the strength of collaboration between authors. Co-cited analysis means that two authors or papers are cited simultaneously by a third author. This study included 23,752 authors and 78,083 co-cited authors. Among them, Stern, Judy E. (64), Luke, Barbara (62), Kissin, Dmitry M. (60), Jamieson, Denise J. (49) and Pinborg, Anja (47) published the most articles, as shown in Table 5). The collaboration between Stern, Judy E. and Kissin, Dmitry M. More was obtained, forming two solid author cooperative groups shown in Figure 6. The study found no collaboration between other authors and the team, and the research is in a relatively scattered state. The results of the co-cited relationship in Table 6 show that Pinborg, A (913), Luke, B (752), Gardner, DK (728), Schieve, La (727) and Hansen, M (611) are the most frequently cited authors. As a result, these authors significantly contribute to ART research.

**Table 5:** Number of articles published by the top 10 authors.

**Rank**	**Author**	**Documents**	**Citations**
1	Stern Judy E.	64	1817
2	Luke Barbara	62	2499
3	Kissin Dmitry M.	60	1954
4	Jamieson Denise J.	49	1960
5	Pinborg Anja	47	1697
6	Boulet Sheree L.	45	1359
7	Esteves Sandro C.	42	1258
8	Qiao Jie	40	654
9	Brown Morton B.	37	1731
10	Bergh Christina	35	1486

**Table 6:** Number of co-citations of the top 10 authors.

**Rank**	**Author**	**Citations**	**Total link strength**
1	Pinborg A	913	25155
2	Luke B	752	15684
3	Gardner DK	728	22944
4	Schieve La	727	16918
5	Hansen M	611	17301
6	Maheshwari A	576	14944
7	Zegers-Hochschild F	537	9884
8	Andersen An	536	11672
9	Kallen B	511	15450
10	La Maraca	453	13026

### Distribution of journals

**Figure 7:**
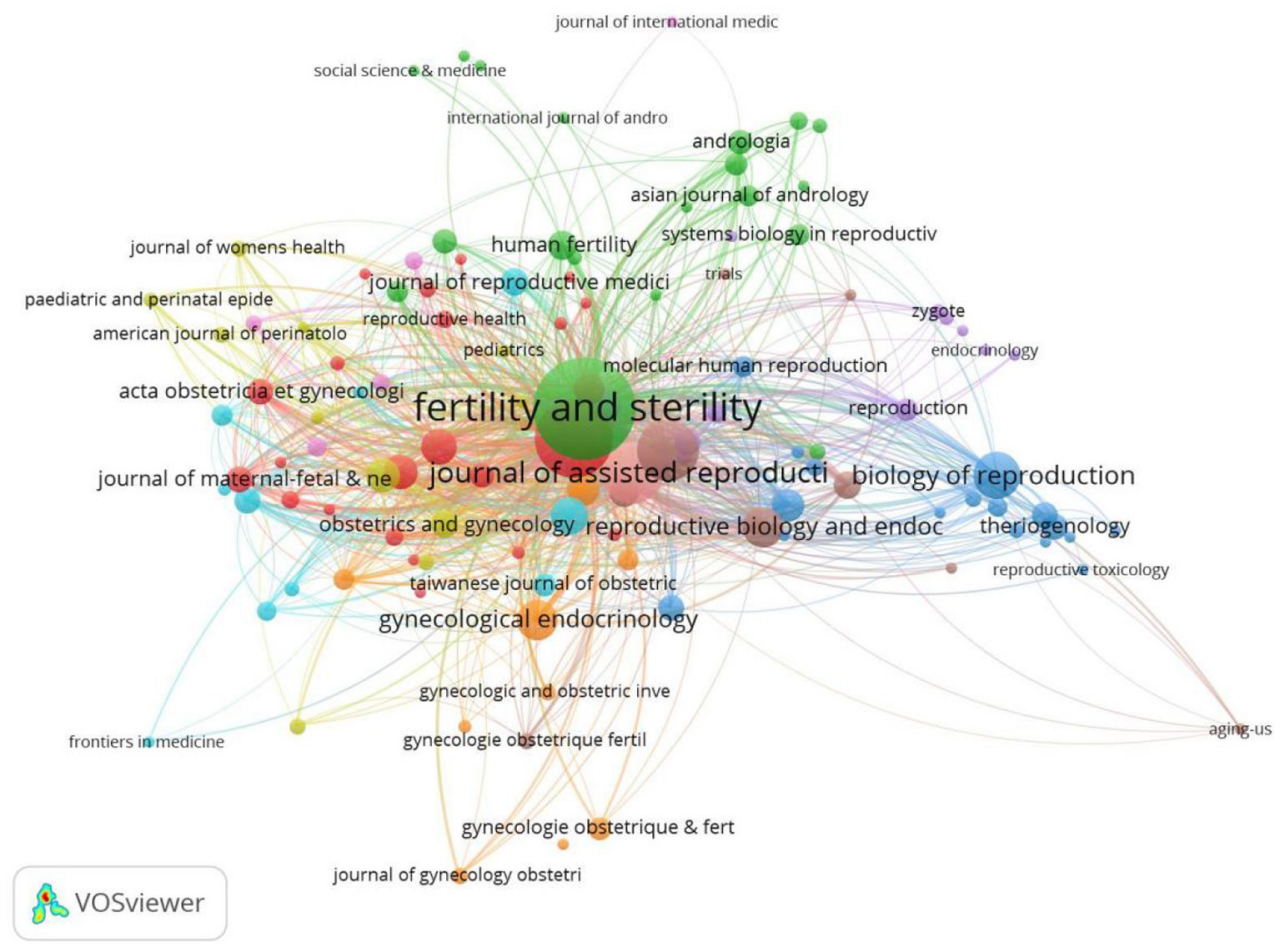
Analysis of cited journals.

The papers used in this study were published in 1,013 journals. The top five assisted journals were Fertility and Sterility (819), Human Reproduction (445), Reproductive Biomedicine Online (273), Journal of Assisted Reproduction and Genetics (269), and Biology of Reproduction (155), as shown in Table 7, Figure 7.

**Table 7:** Top 10 most productive journals.

**Rank**	**Source**	**Documents**	**Citations**	**IF/ JCR (2022)**	**Total link strength**
1	Fertility and Sterility	819	33059	7.490/Q1	7497
2	Human Reproduction	445	23302	6.353/ Q1	5584
3	Reproductive Biomedicine Online	273	5783	4.567/ Q1	1929
4	Journal of Assisted Reproduction and Genetics	269	3476	3.357/ Q2	1825
5	Biology of Reproduction	155	6966	4.161/ Q2	420
6	Reproductive Biology and Endocrinology	112	2066	4.982/ Q1	886
7	Gynecological Endocrinology	111	1360	2.277/ Q3	535
8	Archives of Gynecology and Obstetrics	92	1208	2.493/ Q3	679
9	European Journal of Obstetrics & Gynecology and Reproductive Biology	88	1361	7.413 /Q4	623
10	American Journal of Obstetrics and Gynecology	77	2171	10.693/ Q1	881

The results of the survey on co-cited journals showed that 19,254 journals were co-cited. The top five co-cited journals were Fertil Steril (40105), Hum Reprod (38083), Reprod Biomed Online (8457), Hum Reprod Update (7138) and J Assist Reprod Gen (5325), as shown in Table 8. The top five cited and co-cited journals were divided into Q1 and Q2 subdivisions, reflecting outstanding academic performance in assisted reproductive technology research.

**Table 8:** Top 10 co-cited journals.

**Rank**	**Source**	**Citations**	**Total link strength**
1	Fertil Steril	40105	2102783
2	Hum Reprod	38083	2080856
3	Reprod Biomed Online	8457	570093
4	Hum Reprod Update	7138	481906
5	Jassist Reprod Gen	5325	351433
6	Biol Reprod	5008	427461
7	Obstet Gynecol	4449	236962
8	Am Jobstet Gynecol	4203	250841
9	Lancet	3664	250008
10	Iclin Endocr Metab	3560	334567

### Analysis of highly cited literature and co-cited literature

**Figure 8:**
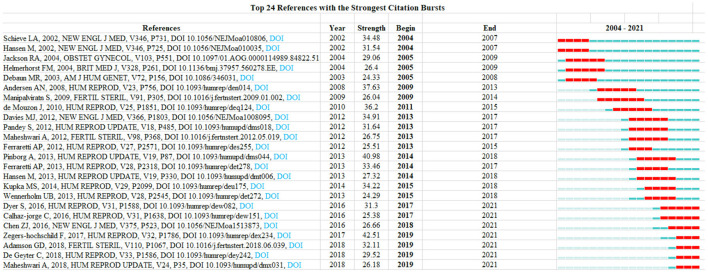
Top 24 References with the strongest citation bursts.

In total, 6286 references and 139023 co-cited references were obtained. The references that exceeded 500 citations include Jirtle and Skinner (13), Jackson et al. (14), Broekmans et al. (15), Zegers-Hochschild et al. (16), Flenady et al. (17), Broekmans et al. (18), LaMarca et al. (19), Zegers-Hochschild et al. (20), Wadhwa et al. (21), Practice Committee of the American Society for Reproductive Medicine (22), and Davies et al. (23), as shown in Table 9. In addition, 24 references were obtained to highlight the analysis results. The three references with the highest intensity were Zegers-Hochschild F, 2017, HUM REPROD, V32, P1786, DOI 10.1093/humrep/dex234 (42.51), Pinborg A, 2013, HUM REPROD UPDATE, V19, P87, DOI 10.1093/humupd/ DMS044 (40.98), Andersen AN, 2008, HUM REPROD, V23, P756, DOI 10.1093/humrep/ DEN014 (37.63), as shown in Figure 8.

**Table 9:** Number of citations of the top 10 references.

**Rank**	**Document**	**Citations**	**Links**
1	Jirtle and Skinner (13)	1642	8
2	Jackson et al. (14)	813	114
3	Broekmans et al. (15)	780	43
4	Zegers-Hochschild et al. (16)	758	37
5	Flenady et al. (17)	734	5
6	Broekmans et al. (18)	594	14
7	La Marca et al. (19)	574	43
8	Zegers-Hochschild et al. (20)	571	39
9	Wadhwa et al. (21)	534	9
10	Practice Committee of the American Society for Reproductive Medicine (22)	526	4

### Keywords analysis

**Figure 9:**
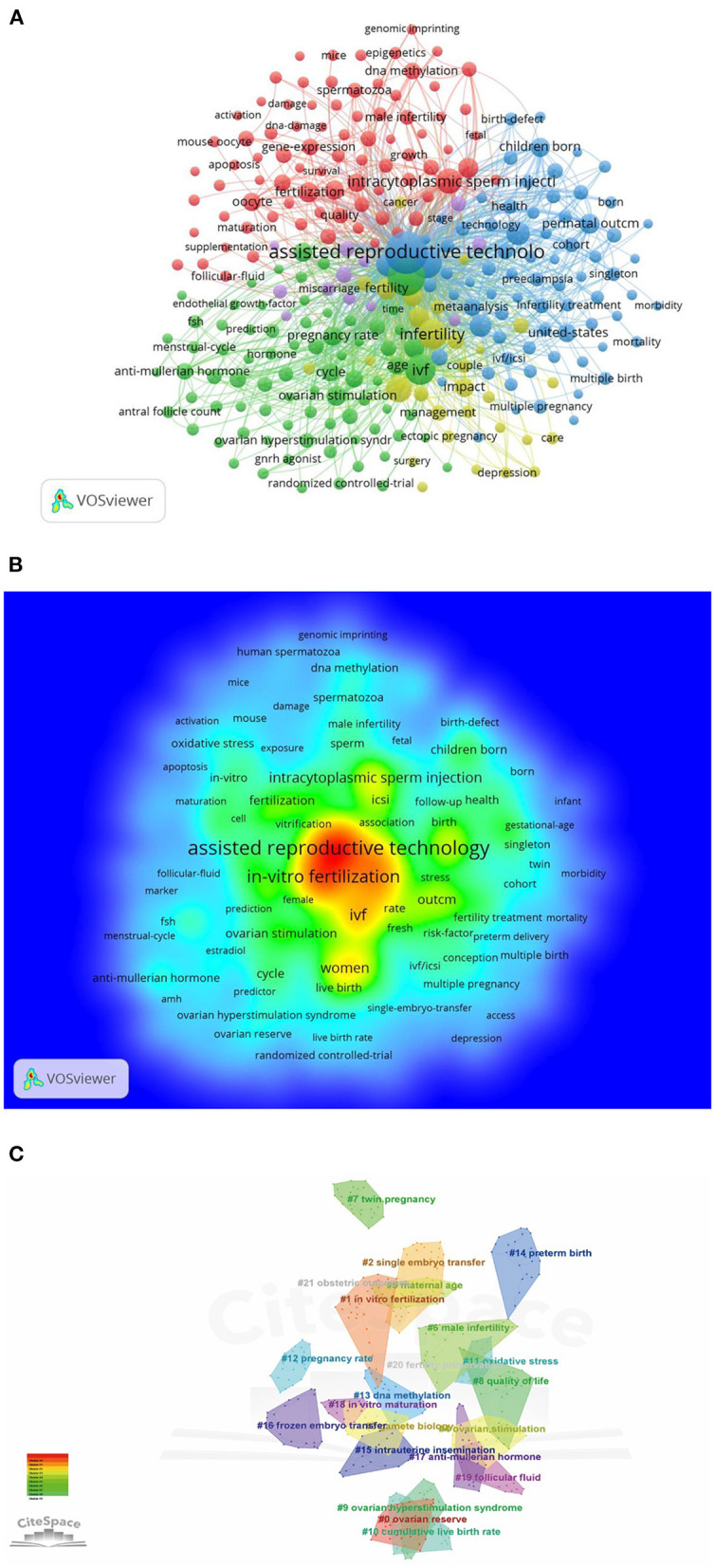
Keywords analysis. **(A)** Keyword co-occurrence analysis map obtained by VOSviewer. The size of the nodes represents the number of occurrences, the thickness of the curve represents the strength of collaboration, and the different colors represent the different clusters. **(B)** Keyword density visualization analysis. The redder the node, the higher the frequency of the keyword. **(C)** Keyword clustering map analysis through CiteSpace. A total of 16 categories of keywords were obtained, and different color blocks represent different keyword clusters.

Through keyword co-occurrence and salience analysis, the changing trend of research topics over time was identified to grasp the development of research hotspots better. In total, 15,417 keywords were obtained. The top ten keywords were In the Assisted reproductive technology (3303), and *in-vitro* were used Fertilization (2139), Ivf (1140), Pregnancy (1140), Women (769), Intracytoplasmic Sperm injection (644), In Fertilization (632), Risk (545), Outcm (423), as shown in Table 10, Figure 9.

**Table 10:** Top 10 keywords on ART.

**Rank**	**Keyword**	**Occurrences**	**Total link strength**
1	Assisted reproductive technology	3303	26817
2	*In-vitro* fertilization	2139	19310
3	Ivf	1140	10552
4	Pregnancy	1140	9678
5	Infertility	1091	8916
6	Women	769	6612
7	Intragytoplasmic Sperm injection	644	5820
8	*In vitro* fertilization	632	5565
9	Risk	545	4749
10	Outcm	423	3570

**Figure 10:**
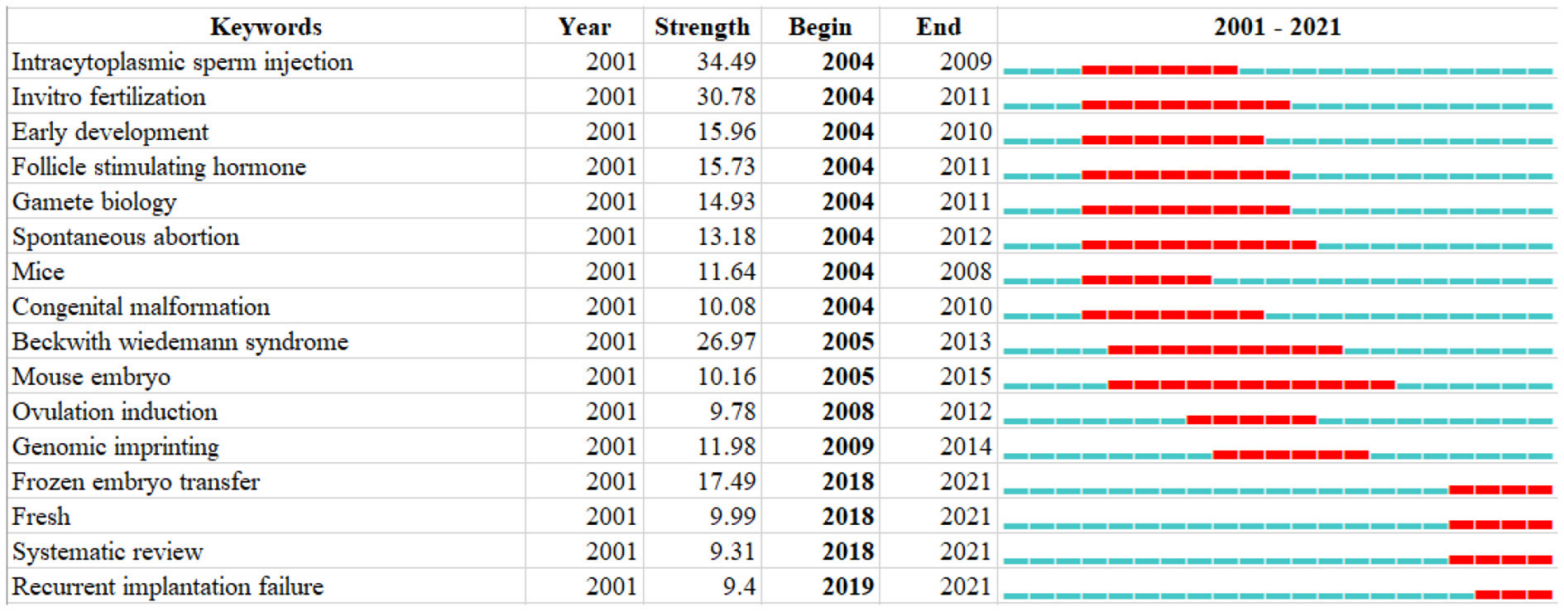
Keywords burst analysis by CiteSpace.

After clustering using the CiteSpace software, 16 keywords were obtained. From 2004 to 2014, research hotspots in ART focused on Intracytoplasmic sperm injection, *in vitro* fertilization, Early development, Follicle-stimulating Hormone, Gamete biology, Spontaneous abortion, Mice, and Congenital malformation. On the other hand, between 2018 and 2021, the research hotspots in ART changed to Frozen embryo transfer, Fresh, Systematic review, and Recurrent implantation failure, as shown in Figure 10.

## Discussion

Scholars can understand the research status of assisted reproduction through a comprehensive and systematic summary of the research topics, research trends and global research status. Assisted reproductive technology (ART) is a common technique to overcome male factor infertility. As a result, recent advances in ART have enabled many infertile couples to have children. Many studies have demonstrated that social factors such as delayed marriage have resulted in more people attending fertility clinics. The studies also state that ART has enabled many older parents to get children. Other studies have stated that the number of people using ART therapy increases yearly (2, 24, 25). ART indications by social change increase the chance of preserving fertility desire and expand, for example, the chances for hope to improve the elderly conception of “social” reasons or for medical reasons (such as saving the oocyte) before the cytotoxic anti-cancer treatment.

With the advent of the era of big data, researchers need to fully understand the developments in their research field. Unlike systematic review or meta-analysis, the bibliometric analysis uses visual software such as VOSviewer and CiteSpace to comprehensively analyze existing literature, to intuitively understand the development trend of research and predict future research hotspots (26). This study is the first to summarize the research status of ART in the past 20 years through bibliometric analysis.

### General information about the literature on assisted reproductive technology

In the past 20 years, the number of studies on ART in journals showed a linear upward trend (R^2^ = 0.9379), especially in the last 4 years, with the annual number of articles published exceeding 500.

From the perspective of countries/regions and institutions, the number of publications and citations of the United States exceeds those of other countries. Although the number of articles published in China ranks second, the number of citations is low, ranking sixth. This finding shows that although the number of papers in China increases yearly, there is still a lack of high-quality articles. This is attributed to the lack of cooperation with international researchers and certain language barriers. Shanghai Jiao Tong Univ is the only institution from Asia among the top 10 organizations with the most published articles, while the rest are from Europe and the United States. Therefore, it is recommended to strengthen communication and cooperation among global cooperative research teams, especially countries and institutions in the Asian region, and look forward to more research results.

This study found that Stern, Judy E. had the highest publication efficiency, and Pinborg, A had the most co-citations, followed by Luke, Barbara, Kissin, Dmitry M., Jamieson, Denise J. Pinborg, and Anja. Stern, Judy E. focuses on intracytoplasmic sperm injection, and Pinborg and A focuses their research on meta-analysis and systematic reviews of ART (27–29).

Related research published in journals is relatively concentrated, with the most published papers followed by other journals. The top five cited and co-cited journals were divided into Q1 and Q2. The study found that most of the papers published are high-quality scientific research achievements.

### Hot spots and frontiers

This study found that the most influential authors and references are review articles and clinical guidelines from internationally renowned institutions and journals. Combined with keyword co-occurrence, clustering and salience analysis, the study identified Intracytoplasmic sperm injection and Frozen embryo transfer as the main research topics and hot spots in ART.

Intracytoplasmic sperm injection, the injection of individual sperm cells directly into the ooplasm, is considered one of the most dramatic technological breakthroughs in ART. Intracytoplasmic sperm injection was introduced in 1992 as a modification of traditional IVF. Currently, ICSI is an established laboratory technique used worldwide to treat infertility. Intracytoplasmic sperm injection was originally introduced to overcome the most severe form of male-factor infertility. Studies have found that although the use of ICSI has steadily increased over the years, the proportion of infertile couples diagnosed with male-factor infertility has remained stable (30). In the more than two decades since its introduction to overcome severe male factor infertility, ICSI has been widely used to treat both male and non-male factor infertility. However, the advantage of ICSI over traditional IVF in couples without male factor infertility has not been demonstrated (31).

When performing ART in humans, sperm head morphology, size, and acrosome are important criteria for sperm selection (32), as the size of the sperm head may affect the fertilization rate (33). In contrast, Zahiri and Ghasemian reported that acrosome size and morphology of sperm heads influence sperm chromatin status, fertilization rates, and clinical outcomes (34, 35). Unlike sperm with normal acrosomes, sperm with small or large acrosomes significantly lower fertilization rate. In addition, sperm heads with large acrosomes reduce implantation rates, clinical pregnancy, and live birth rates. Many studies have reported that patients with spherospermia and abnormal acrosomes have significantly higher DNA fragmentation, sex chromosome aneuploidy, and disomy compared than the controls (36). In addition, hidden defects in normal-looking sperm may be responsible for the failed fertilization, suggesting the need for simple routine tests to detect these defects (37).

In a prospective study involving 1,089 randomly selected sibling oocytes during ART cycles in patients with polycystic ovary syndrome (PCOS), fertilization rates and embryo development were compared between C-IVF and ICSI in PCOS patients as a sole indication of infertility. The results showed a higher fertilization rate in the ICSI group (73%) than in the C-IVF group (45%) (38). In one case, after 6 weeks of treatment with 1200 mg of d-chiro-inositol (DCI), ovulation resumed in two non-PCOS anovatory women with elevated progesterone and luteinizing hormone and endometrial thickening (39).

The frequency of frozen embryo transfer (FET) continues to increase worldwide due to improved embryo survival through the introduction of vitrification, the implementation of guidelines to promote single-embryo transfer and therefore increased cryopreservation of excess embryos, efforts to reduce ovarian hyperstimulation rate syndrome (OHSS), the use of preimplantation genetic testing, and increased cryopreservation of embryos for fertility preservation (40–43).

*In vitro* fertilization laboratories quickly adopted vitrification after its efficiency was revealed in several publications on oocyte cryopreserve. The technique has also been rapidly adopted for embryo cryopreservation and is now the gold standard worldwide (44–46). The three most significant benefits of embryo vitrification are increased embryo survival (maintenance of viability) which increases the efficiency of embryo transfer/IVF treatment, increased cumulative pregnancy rate, and improved safety of assisted reproduction. Vitrification has directly contributed to the widespread acceptance of elective single-embryo transfer resulting in a sharp decline in the incidence of twins and higher rates of multiple pregnancies with IVF treatment.

Vitrification is a breakthrough in ARP since it revolutionized how IVF patients are treated and managed. In addition, vitrification has opened up new options for patients, most notably fertility preservation (*via* oocyte cryopreservation) and donor egg banking. The fact that vitrification has similar or even better results than fresh embryo transfer on some indicators makes it possible to abandon fresh embryo transfer altogether in favor of freezing all methods; Embryo biopsy (and preimplantation genetic testing) without compromising embryo survival; Elective single embryo transfer (and maintaining a high pregnancy rate); And significantly improved the single transplant cycle and cumulative pregnancy/live birth rate.

In recent years, uterine transplantation (UTX) has enabled women suffering from absolute uterine factor infertility (AUFI) to give birth to biological children and as an alternative to surrogacy. In addition, advances in techniques such as tissue engineering are expected to address UTX-related complications and difficulties in organ supply (47). Besides, in the past few years, a new branch of medicine with distinct multidisciplinary characteristics has developed: tumor fertility, which has attracted more and more attention. Maintaining fertility and family planning are key issues that must be addressed in all cancer patients of reproductive age (48).

According to some studies (49–52), infants born after FET have a lower risk of preterm birth that infants born after fresh embryo transfer. In addition, many studies have reported that infants born after FET have a lower risk of being small for gestational age and a higher birth weight than infants born after fresh embryo transfer (53–56). Of course, given that large-scale implementation of ART is relatively recent, further research is needed to provide more conclusive evidence on outcomes and impacts (57).

ART is a valuable option for couples who are infertile or have fertility problems.At the same time, ART, as an important part of the so-called “reproductive revolution,” has brought about three related results: the rift between reproduction and sexual intercourse, the opportunity to use heterologous fertilization through donor gametes, and the consequent increase in the number of reproductive donors (58). Therefore, some disputes in the legal and ethical aspects of ART need further consensus (59–62). With the outbreak of the COVID-19 pandemic, some experts have pointed out that access to assisted fertilization technology for infertile couples should be a part of the right to health, rather than a right to parenthood or an increase in birth rates (63).

This study found that the development of ART research areas is not balanced as influential authors and institutions are concentrated in Europe and Asia. This is attributed to the fact that ART is needed more in Europe and Asia than in most parts of the world (24, 64, 65). The study also found that the mechanism of ART and the pregnancy and live birth rate need to be further improved.

### Limitations and prospects

Although this study is the first bibliometric analysis of research on ART in the last 20 years, it has some limitations. First, most high-quality articles in recent years have not reached the ideal citation time, which is prone to research bias. Second, there may be a time delay in exploring the frontiers of research. Lastly, this study included English literature retrieved from the WOS database. As a result, the likelihood of omitting quality articles published in other languages is high.

## Conclusion

The bibliometric analysis of this study provides comprehensive information on the publication of ART research papers in various journals. The results found that ART is booming and has aroused great interest in the research community worldwide. Although ART is still in its infancy, there is great potential to trigger the development of ART. This study concludes that future research on Frozen embryo transfer could be at the forefront of assisted reproduction.

## Data availability statement

The original contributions presented in the study are included in the article/supplementary material, further inquiries can be directed to the corresponding authors.

## Author contributions

FM and JW designed the study. LW, YZ, MZ, HL, GG, and DL conducted the literature search. FM, SD, and LW analyzed the data and wrote the paper. JW and XL approved the final manuscript. All authors contributed to the article and approved the submitted version.

## Funding

This work was supported by the China Postdoctoral Innovative Talent Support Program (BX20220047), Young Talent Support Project of Beijing Association of Science and Technology (BYESS2022182), and Young Talent Support Project of Chinese Association of Chinese Medicine (CACM-2021-QNRC2-B04).

## Conflict of interest

The authors declare that the research was conducted in the absence of any commercial or financial relationships that could be construed as a potential conflict of interest.

## Publisher's note

All claims expressed in this article are solely those of the authors and do not necessarily represent those of their affiliated organizations, or those of the publisher, the editors and the reviewers. Any product that may be evaluated in this article, or claim that may be made by its manufacturer, is not guaranteed or endorsed by the publisher.

## References

[1] Del Giudice F Kasman AM Chen T De Berardinis E Busetto GM Sciarra A. The Association between mortality and male infertility: systematic review and meta-analysis. Urology. (2021) 154:148–57. 10.1016/j.urology.2021.02.041 33819517

[2] De Geyter C Calhaz-Jorge C Kupka MS Wyns C Mocanu E Motrenko T. ART in Europe, 2014: results generated from European registries by ESHRE: The European IVF-monitoring Consortium (EIM) for the European Society of Human Reproduction and Embryology (ESHRE). Hum Reprod. (2018) 33:1586–601. 10.1093/humrep/dey242 30032255

[3] Pritchard A. Statistical bibliography or bibliometrics. J Document. (1969) 25:348–9. 10.1108/eb026482

[4] Deng Z Wang H Chen Z Wang T. Bibliometric Analysis of Dendritic Epidermal T Cell (DETC) research from 1983 to 2019. Front Immunol. (2020) 11:259. 10.3389/fimmu.2020.00259 32226424 PMC7080701

[5] Wang S Zhou H Zheng L Zhu W Zhu L Feng D. Global trends in research of macrophages associated with acute lung injury over past 10 years: a bibliometric analysis. Front Immunol. (2021) 12:669539. 10.3389/fimmu.2021.669539 34093568 PMC8173163

[6] Moral-Muñoz JA Herrera-Viedma E Santisteban-Espejo A Cobo MJ. Software tools for conducting bibliometric analysis in science: an up-to-date review. El Prof Inf. (2020) 29. 10.3145/epi.2020.ene.03

[7] Chen C. Searching for Intellectual turning points: progressive knowledge domain visualization. Proc Natl Acad Sci U S A. (2004) 101:5303–10. 10.1073/pnas.0307513100 14724295 PMC387312

[8] Yao L Hui L Yang Z Chen X Xiao A. Freshwater microplas- tics pollution: detecting and visualizing emerging trends based on Citespace II. Chemosphere. (2020) 245:125627. 10.1016/j.chemosphere.2019.125627 31864046

[9] Paolino L Pravettoni R Epaud S Ortala M Lazzati A. Comparison of surgical activity and scientific publications in bariatric surgery: an epidemiological and bibliometric analysis. ObesSurg. (2020) 30:3822–30. 10.1007/s11695-020-04703-0 32451915

[10] Chen T Zhu J Zhao Y Li H Li P Fan J. The global state of research in pain management of osteoarthritis (2000–2019): a 20-year visualized analysis. Medicine (Baltimore). (2021) 100:e23944. 10.1097/MD.0000000000023944 33466135 PMC7808549

[11] Chen C. CiteSpace II: detecting and visualizing emerging trends and transient patterns in scientific literature. J Am Soc Inf Sci. (2006) 57:359–77. 10.1002/asi.20317

[12] Van Eck NJ Waltman L. Software survey: VOSviewer, a computer program for bibliometric mapping. Scientometrics. (2010) 84:523–38. 10.1007/s11192-009-0146-3 20585380 PMC2883932

[13] Jirtle R Skinner M. Environmental epigenomics and disease susceptibility. Nat Rev Genet. (2007) 8:253–62. 10.1038/nrg2045 17363974 PMC5940010

[14] Jackson RA Gibson KA Wu YW Croughan MS. Perinatal outcomes in singletons following *in vitro* fertilization: A meta-analysis. Obstet Gynecol. (2004) 103:551–63. 10.1097/01.AOG.0000114989.84822.51 14990421

[15] Broekmans FJ Kwee J Hendriks DJ Mol BW Lambalk CB. A systematic review of tests predicting ovarian reserve and IVF outcome. Hum Reprod Update. (2006) 12:685–718. 10.1093/humupd/dml034 16891297

[16] Zegers-Hochschild F Adamson GD de Mouzon J Ishihara O Mansour R Nygren K. International Committee for Monitoring Assisted Reproductive Technology (ICMART) and the World Health Organization (WHO) revised glossary of ART terminology, 2009. Fertil Steril. (2009) 92:1520–4. 10.1016/j.fertnstert.2009.09.009 19828144

[17] Flenady V Koopmans L Middleton P Frøen JF Smith GC Gibbons K. Major risk factors for stillbirth in high-income countries: a systematic review and meta-analysis. Lancet. (2011) 377:1331–40. 10.1016/S0140-6736(10)62233-7 21496916

[18] Broekmans FJ Soules MR Fauser BC. Ovarian aging: mechanisms and clinical consequences. Endocr Rev. (2009) 30:465–93. 10.1210/er.2009-0006 19589949

[19] La Marca A Sighinolfi G Radi D Argento C Baraldi E Artenisio AC. Anti-Mullerian hormone (AMH) as a predictive marker in assisted reproductive technology (ART). Hum Reprod Update. (2010) 16:113–30. 10.1093/humupd/dmp036 19793843

[20] Zegers-Hochschild F Adamson GD de Mouzon J Ishihara O Mansour R Nygren K. The International Committee for Monitoring Assisted Reproductive Technology (ICMART) and the World Health Organization (WHO) Revised Glossary on ART Terminology, 2009. Hum Reprod. (2009) 24:2683–7. 10.1093/humrep/dep343 19801627

[21] Wadhwa PD Buss C Entringer S Swanson JM. Developmental origins of health and disease: brief history of the approach and current focus on epigenetic mechanisms. Semin Reprod Med. (2009) 27:358–68. 10.1055/s-0029-1237424 19711246 PMC2862635

[22] Practice Committee of the American Society for Reproductive Medicine. Evaluation and treatment of recurrent pregnancy loss: a committee opinion. Fertil Steril. (2012) 98:1103–11. 10.1016/j.fertnstert.2012.06.048 22835448

[23] Davies MJ Moore VM Willson KJ Van Essen P Priest K Scott H. Reproductive technologies and the risk of birth defects. N Engl J Med. (2012) 366:1803–13. 10.1056/NEJMoa1008095 22559061

[24] Calhaz-Jorge C De Geyter C Kupka MS de Mouzon J Erb K Mocanu E. Assisted reproductive technology in Europe, 2013: results generated from European registers by ESHRE. Hum Reprod. (2017) 32:1957–73. 10.1093/humrep/dex264 29117383

[25] Sunderam SK Kissin DM Zhang Y Jewett A Boulet SL Warner L. Assisted Reproductive Technology Surveillance–United States, 2017. MMWR Surveill Summ. (2020) 69:1–20. 10.15585/mmwr.ss6909a1 33332294 PMC7755269

[26] Keeffe EB O'Connor KW. 1989 A/S/G/E survey of endoscopic sedation and monitoring practices. Gastrointest Endosc. (1990) 36:S13–8. 2351253

[27] Söderström-Anttila V Wennerholm UB Loft A Pinborg A Aittomäki K Romundstad LB. Surrogacy: outcomes for surrogate mothers, children and the resulting families-a systematic review. Hum Reprod Update. (2016) 22:260–76. 10.1093/humupd/dmv046 26454266

[28] Wolf HT Huusom LD Henriksen TB Hegaard HK Brok J Pinborg A. Magnesium sulphate for fetal neuroprotection at imminent risk for preterm delivery: a systematic review with meta-analysis and trial sequential analysis. BJOG. (2020) 127:1180–8. 10.1111/1471-0528.16238 32237069

[29] Pinborg A Loft A Romundstad LB Wennerholm UB Söderström-Anttila V Bergh C. Epigenetics and assisted reproductive technologies. Acta Obstet Gynecol Scand. (2016) 95:10–5. 10.1111/aogs.12799 26458360

[30] Jain T Gupta R. T rends in the use of intracytoplasmic sperm injection in the United States. N Engl J Med. (2007) 357, 251–57. 10.1056/NEJMsa070707 17634460

[31] Esteves SC Roque M Bedoschi G Haahr T Humaidan P. Intracytoplasmic sperm injection for male infertility and consequences for offspring. Nat Rev Urol. (2018) 15:535–62. 10.1038/s41585-018-0051-8 29967387

[32] Itoi F Miyamoto T Himaki T Honnma H Sano M Ueda J. Importance of real-time measurement of sperm head morphology in intracytoplasmic sperm injection. Zygote. (2022) 30:9–16. 10.1017/S0967199421000307 33988119

[33] Nishikawa K Itoi F Nagahara M Jose M Matsunaga A Ueda J and Iwamoto T. The normality of sperm in an infertile man with ring chromosome 15: a case report. J Assist Reprod Genet. (2018) 35:251–6. 10.1007/s10815-017-1061-9 29063501 PMC5845033

[34] Zahiri Z and Ghasemian F. Is it necessary to focus on morphologically normal acrosome of sperm during intracytoplasmic sperm injection? Indian J Med Res. (2019) 150:477–85. 10.4103/ijmr.IJMR_866_18 31939391 PMC6977361

[35] Ghasemian F Mirroshandel SA Monji-Azad S Azarnia M Zahiri Z. An efficient method for automatic morphological abnormality detection from human sperm images. Comput Methods Programs Biomed. (2015) 122:409–20. 10.1016/j.cmpb.2015.08.013 26345335

[36] Brahem S Mehdi M Elghezal H Saad A. Analysis of sperm aneuploidies and DNA fragmentation in patients with globozoospermia or with abnormal acrosomes. Urology. (2011) 77:1343–8. 10.1016/j.urology.2010.12.015 21310465

[37] Goudakou M Kalogeraki A Matalliotakis I Panagiotidis Y Gullo G Prapas Y. Cryptic sperm defects may be the cause for total fertilization failure in oocyte donor cycles. Reprod Biomed Online. (2012) 24:148–52. 10.1016/j.rbmo.2011.10.011 22197604

[38] Hwang JL Seow KM Lin YH Hsieh BC Huang LW Chen HJ. IVF versus ICSI in sibling oocytes from patients with polycystic ovarian syndrome: a randomized controlled trial. Hum Reprod. (2005) 20:1261–65. 10.1093/humrep/deh786 15705619

[39] Bezerra Espinola MS Laganà AS Bilotta G Gullo G Aragona C Unfer V. D-chiro-inositol Induces Ovulation in Non-Polycystic Ovary Syndrome (PCOS), non-insulin-resistant young women, likely by modulating aromatase expression: a report of 2 cases. Am J Case Rep. (2021) 22:e932722. 10.12659/AJCR.932722 34615846 PMC8503791

[40] Niederberger C Pellicer A Cohen J Gardner DK Palermo GD O'Neill CL. Forty years of IVF. Fertil Steril. (2018) 110:185–324 e5. 10.1016/j.fertnstert.2018.06.005 30053940

[41] Banker M Dyer S Chambers GM Ishihara O Kupka M de Mouzon J. International Committee for Monitoring Assisted Reproductive Technologies (ICMART): world report on assisted reproductive technologies, 2013. Fertil Steril. (2021) 116:741–56. 10.1016/j.fertnstert.2021.03.039 33926722

[42] Practice Practice Committee of the American Society for Reproductive M the Practice Committee for the Society for Assisted Reproductive Technologies. Electronic address AAO. Guidance on the limits to the number of embryos to transfer: a committee opinion. Fertil Steril. (2021) 116:651–4. 7. 10.1016/j.fertnstert.2021.06.050 34330423

[43] Donnez J Dolmans MM. Fertility preservation in men and women: where are we in 2021? Are we rising to the challenge? Fertil Steril. (2021) 115:1089–90 10.1016/j.fertnstert.2021.03.028 33823991

[44] Chang CC Shapiro DB Bernal DP Wright G Kort HI Nagy ZP. Human oocyte vitrification: *in-vivo and in-vitro* maturation outcomes. Reprod Biomed Online. (2008) 17:684–8. 10.1016/S1472-6483(10)60316-1 18983753

[45] Cobo A Kuwayama M Perez S Ruiz A Pellicer A Remohi J. Comparison of concomitant outcome achieved with fresh and cryopreserved donor oocytes vitrified by the Cryotop method. Fertil Steril. (2008) 89:1657–64. 10.1016/j.fertnstert.2007.05.050 17889865

[46] Kuwayama M Vajta G Kato O Leibo SP. Highly efficient vitrification method for cryopreservation of human oocytes. Reprod Biomed Online. (2005) 11:300–8. 10.1016/S1472-6483(10)60837-1 16176668

[47] Gullo G Etrusco A Fabio M Cucinella G Rossi C Billone V. The reproductive potential of uterus transplantation: future prospects. Acta Biomed. (2022) 93:e2022138. 10.23750/abm.v93i2.12868 35546000 PMC9171877

[48] Zaami S Melcarne R Patrone R Gullo G Negro F Napoletano G. Oncofertility and reproductive counseling in patients with breast cancer: a retrospective study. J Clin Med. (2022) 11:1311. 10.3390/jcm11051311 35268402 PMC8911138

[49] Pinborg A Loft A Aaris Henningsen AK Rasmussen S Andersen AN. Infant outcome of 957 singletons born after frozen embryo replacement: the Danish National Cohort Study 1995–2006. Fertil Steril. (2010) 94:1320–7. 10.1016/j.fertnstert.2009.05.091 19647236

[50] Shih W Rushford DD Bourne H Garrett C McBain JC Healy DL. Factors affecting low birthweight after assisted reproduction technology: difference between transfer of fresh and cryopreserved embryos suggests an adverse effect of oocyte collection. Hum Reprod. (2008) 23:1644–53. 10.1093/humrep/den150 18442997

[51] Pelkonen S Gissler M Koivurova S Lehtinen S Martikainen H Hartikainen AL. Physical health of singleton children born after frozen embryo transfer using slow freezing: a 3-year follow-up study. Hum Reprod. (2015) 30:2411–8. 10.1093/humrep/dev203 26293785

[52] Wennerholm UB Henningsen AK Romundstad LB Bergh C Pinborg A Skjaerven R. Perinatal outcomes of children born after frozen-thawed embryo transfer: a Nordic cohort study from the CoNARTaS group. Hum Reprod. (2013) 28:2545–53. 10.1093/humrep/det272 23832793

[53] Maheshwari A Raja EA Bhattacharya S. Obstetric and perinatal outcomes after either fresh or thawed frozen embryo transfer: an analysis of 112,432 singleton pregnancies recorded in the human fertilisation and embryology authority anonymized dataset. Fertil Steril. (2016) 106:1703–8. 10.1016/j.fertnstert.2016.08.047 27678031

[54] Vidal M Vellve K Gonzalez-Comadran M Robles A Prat M Torne M. Perinatal outcomes in children born after fresh or frozen embryo transfer: a Catalan cohort study based on 14,262 newborns. Fertil Steril. (2017) 107:940–7. 10.1016/j.fertnstert.2017.01.021 28292612

[55] Belva F Bonduelle M Roelants M Verheyen G V an Landuyt L. Neonatal health including congenital malformation risk of 1072 children born after vitrified embryo transfer. Hum Reprod. (2016) 31:1610–20. 10.1093/humrep/dew103 27165622

[56] Bosch E De Vos M Humaidan P. The future of cryopreservation in assisted reproductive technologies. Front Endocrinol. (2020) 11:67. 10.3389/fendo.2020.00067 32153506 PMC7044122

[57] Gullo G Scaglione M Cucinella G Chiantera V Perino A Greco ME. Neonatal outcomes and long-term follow-up of children born from frozen embryo, a narrative review of latest research findings. Medicina (Kaunas). (2022) 58:1218. 10.3390/medicina58091218 36143894 PMC9500816

[58] Negro F Marinelli S. Is there anything left of the Italian law governing medically-assisted procreation? Clin Ter. (2021) 171:e57–9. 10.7417/CT.2021.2283 33346329

[59] Zaami S. Assisted heterologous fertilization and the right of donorconceived children to know their biological origins. Clin Ter. (2018) 169:e39–43. 10.7417/T.2018.2052 29446790

[60] Ethics Committee of the American Society for Reproductive Medicine. Electronic address: asrm@asrm.org; Ethics Committee of the American Society for Reproductive Medicine. Misconduct in third-party assisted reproduction: an ethics committee opinion. Fertil Steril. (2018) 110:1012–16. 10.1016/j.fertnstert.2018.08.030 30396537

[61] Montanari Vergallo G Marinelli E di Luca NM Zaami S. Gamete donation: are children entitled to know their genetic origins? A Comparison of opposing views the Italian State of affairs. Eur J Health Law. (2018) 25:322–37. 10.1163/15718093-12530378

[62] Ghoshal R. Assisted reproductive technologies: conundrums and challenges. Indian J Med Ethics. (2018) 3:95–8. 10.20529/IJME.2018.030 29724695

[63] Rallo G Negro F Consalvo F Piersanti V Marinelli S. Medically assisted procreation in times of COVID-19: what impact on health care system organization and the reproductive rights of couples? Acta Biomed. (2021) 92:e2021275. 10.23750/abm.v92i5.11900 34738563 PMC8689323

[64] Calhaz-Jorge C De Geyter CH Kupka MS Wyns C Mocanu E Motrenko T. Survey on ART and IUI: legislation, regulation, funding and registries in European countries: The European IVF-monitoring Consortium (EIM) for the European Society of Human Reproduction and Embryology (ESHRE). Hum Reprod Open. (2020) 2020:hoz044. 10.1093/hropen/hoz044 32042927 PMC7002185

[65] Irahara M Kuwahara A Iwasa T Ishikawa T Ishihara O. Assisted reproductive technology in Japan: a summary report of 1992–2014 by the Ethics Committee, Japan Society of Obstetrics and Gynecology. Reprod Med Biol. (2017) 16:126–32. 10.1002/rmb2.12014 29259459 PMC5661813

